# The experience of primary care and the institutionalization of health evaluation in Brazil: necessary advances

**DOI:** 10.1590/0102-311XEN214326

**Published:** 2026-11-09

**Authors:** Eronildo Felisberto, Gabriella de Almeida Raschke Medeiros, Isabella Samico, Zulmira Maria de Araújo Hartz, Luciana Santos Dubeux, Ana Coelho de Albuquerque, Juliana Martins Barbosa da Silva Costa

**Affiliations:** 1 Grupo de Estudos em Gestão e Avaliação em Saúde, Instituto de Medicina Integral Prof. Fernando Figueira, Recife, Brasil.; 2 Secretaria de Atenção Primária à Saúde, Ministério da Saúde, Brasília, Brasil.; 3 Centro Acadêmico do Agreste, Universidade Federal de Pernambuco, Caruaru, Brasil.

We thank the commentators for keeping the debate open and purposeful so that it raises new stances from researchers, professionals, and managers. Your comments develop the core issues of the article ^1^ and help to shift the debate away from why to how, where, by whom, and for what to institutionalize. This is relevant because it shows that the consolidation of an evaluation policy stem from the capacity to produce sustainable practices distributed across the levels of the system and in connection to social needs rather than only resulting from its inscription in the structures of the State.

Matthew Harris’ ^2^ distinction between institutionalizing evaluations of and by primary health care (PHC) is thought-provoking. The former refers to policies, structures, financing, information systems, and evaluations that can answer questions about PHC performance and effects. The latter focuses on teams, managers, and communities, questioning their ability to understand needs, critically analyze information, and produce knowledge about their own practices, including their relationship with other levels of care. Rather than a question of choosing between these perspectives, institutionalization only acquires density when both meet.

This distinction makes explicit a recurring problem: producing data fails to mean promoting evaluation. The experience of PHC has expanded the availability of data, as Marly M. Cruz ^3^ points out, but its transformation into intelligence remains uneven. As Harris ^2^ notes, an evaluative culture presupposes curiosity, methodological capacity, and willingness to question results and modify practices. Thus, institutionalizing evaluation requires investing in national and subnational structures and in evaluative capacity, incorporating training in evaluation in permanent education, residencies, training of community agents and other professionals, and an evaluative research agenda that addresses the identified gaps.

This argument finds resonance in Ana Luiza d’Ávila Viana’s ^4^ contribution: a policy to evaluate a multilevel system must also be multilevel. Although we have highlighted tripartite governance as an attribute of a Brazilian National Evaluation Policy, Viana ^4^ urges us forward toward the necessary construction of a federative architecture of evaluation that negotiates responsibilities, resources, capacities, and uses across levels of government in articulation with instances of agreement and social control.

This implies acknowledging that institutionalization is unable to be conceived as a linear process of guideline diffusion from the federal level to the territories. Federated entities have profoundly unequal institutional capacities, whereas regions, municipalities, and teams face varying needs. Permanent governance instances, the participation of universities and collaborative networks, dissemination of results, and mechanisms of interfederative cooperation can reduce this asymmetry and transform evaluation into a shared practice of governance.

Here one must highlight the incongruity between the *Ministerial Ordinances GM/MS n. 3,201* ^5^ and *n. 8,585* ^6^, which address the establishment of the Monitoring and Evaluation Unit of the Brazilian Unified National Health System (CCMA-SUS, acronym in Portuguese) and the Brazilian Ministry of Health Monitoring and Evaluation Policy (PMA, acronym in Portuguese), respectively. Although one of them (despite its internal inconsistency) refers to the SUS, both disregard its interfederative and tripartite character. If this assumption is correct, it is necessary to correct the governance of these norms signaling a limited reading of the Organic Health Law. It seems to us that its fulfillment (and in respect for the constitutional principles of integrality, decentralization, and social participation) requires reviewing the committee composition (solely composed of Brazilian Ministry of Health members) and the alignment of the scope of these regulations. On the other hand, the constitutional guidelines clearly signal the need for an updated look at the changes in the health system and society in the almost 40 years after their formulation for they require a PMA for the SUS with a tripartite agreement and submission to social control.

However, we can and must go further. Luis Augusto Coelho Pisco’s ^7^ contribution introduces another necessary frontier. If evaluation is part of a democratic governance architecture, its institutionalization must consider the public-private configuration of the Brazilian health system. A policy committed to universality and equity must ask what and how to evaluate and who is evaluated by whom and under what public responsibilities. The asymmetry between evaluation and accountability mechanisms applied to the SUS and those levied on providers and private organizations can produce a disproportionate accountability of the public sector. Broadening this discussion means situating evaluation within the regulation of the system itself and reaffirming that private interests interfering in the conditions of access, continuity, and integrality must also belong to the evaluation visibility field.

This question refers to the paradox we have discussed: institutionalization strengthens evaluation, but can simultaneously domesticate it ^1^. Indicators, goals, certifications, and financial incentives can favor transparency and accountability, but also convert evaluation into a bureaucratic device of compliance or legitimization of previously established decisions. Thus, institutionalization also implies creating conditions for evaluation independence, plurality, and critical capacity, ensuring transparency, social participation, approach diversity, funding stability, and public spaces to discuss results so that they effectively influence decisions.

Thus, Cruz’s ^4^ reflection gains strength about digital transformation and the expansion of the capacity to produce information. While this context might favor Pisco’s ^7^ proposal, unequal infrastructure, low interoperability, and the bureaucratic use of information systems could reproduce or exacerbate inequalities. The central question is no longer how much data is produced, but what knowledge we produce and for whom and with what consequences. New technologies should strengthen learning, social participation, and equity-driven decisions rather than increasing the capacity for measurement and control.

At this point also lies resilience, highlighted by Viana ^4^. More than the ability to return to the state prior to a crisis, we are interested in a resilience that can produce learning and transformation. Climate change, health emergencies, violence, racial and social inequalities, and precarious living challenge evaluations focused exclusively on service performance. Evaluating requires understanding how systems, institutions, and territories respond, learn, and transform themselves in the face of these processes.

The contributions of the debaters can more concretely outline the policy the need of which we defend - that would entail articulating federative governance, evaluative capacity, production monitoring and intelligence, accountability, learning, institutional stability, critical independence, digital transformation and equity, and scientific and social knowledge. Perhaps, this articulation holds the deepest meaning of institutionalization. We want evaluation to not simply occupy another place in the State bureaucracy, but that it becomes a permanent capacity of the Brazilian health system to question itself, dialogue with society, learn from its territories, and transform its practices. In this perspective, institutionalization, rather than meaning stabilizing the evaluation to avoid changes, entails creating conditions for it to remain stable enough to continue producing changes.

## Data Availability

Not applicable.

## References

[1] Felisberto E, Medeiros GAR, Samico I, Hartz ZMA, Dubeux LS, Albuquerque AC (2026). Evaluation in primary health care in Brazil reflections on institutionalization. Cad Saúde Pública.

[2] Harris M (2026). Institutionalizing evaluation "of" or "by" primary care different processes, equal importance. Cad Saúde Pública.

[3] Cruz M (2026). Perspectives and controversies surrounding strategies for institutionalizing primary health care evaluation within the Brazilian Unified National Health System. Cad Saúde Pública.

[4] Viana ALd'A (2026). A multilevel look at the construction of the monitoring and evaluation policy.. Cad Saúde Pública.

[5] Ministério da Saúde (2020). Portaria GM/MS nº 3.201, de 27 de novembro de 2020. Institui o Comitê Consultivo de Monitoramento e Avaliação do Sistema Único da Saúde (CCMA-SUS) no âmbito do Ministério da Saúde.. Diário Oficial da União.

[6] Ministério da Saúde (2025). Portaria GM/MS nº 8.585, de 29 de outubro de 2025. Institui, no âmbito do Ministério da Saúde, a Política de Monitoramento e Avaliação - PMA-MS, e altera a Portaria GM/MS nº 3.201, de 27 de novembro de 2020, para atualizar as competências do Comitê Consultivo de Monitoramento e Avaliação do Sistema Único de Saúde - CCMA-SUS.. Diário Oficial da União.

[7] Pisco LAC (2026). Institutionalization of evaluation in primary health care in Brazil trajectory, advances, and contemporary challenges. Cad Saúde Pública.

