# Supplementary material for: The experience of primary care and the institutionalization of health evaluation in Brazil: necessary advances
Source: Cad Saude Publica. 2026 Nov 9;42(Suppl 2):e00214326. doi: 10.1590/0102-311XEN214326 (PMC13750640; doi:10.1590/0102-311XEN214326)
Supplement: Translation

# Translation: A experiência da atenção primária e a institucionalização da avaliação em saúde no Brasil: avanços necessários

**DOI:** 10.1590/0102-311XPT214326

**Authors:** Eronildo Felisberto, Gabriella de Almeida Raschke Medeiros, Isabella Samico, Zulmira Maria de Araújo Hartz, Luciana Santos Dubeux, Ana Coelho de Albuquerque, Juliana Martins Barbosa da Silva Costa

**Affiliations:** 1 Grupo de Estudos em Gestão e Avaliação em Saúde, Instituto de Medicina Integral Prof. Fernando Figueira, Recife, Brasil.; 2 Secretaria de Atenção Primária à Saúde, Ministério da Saúde, Brasília, Brasil.; 3 Centro Acadêmico do Agreste, Universidade Federal de Pernambuco, Caruaru, Brasil.

Agradecemos aos comentaristas por manter o debate aberto e propositivo, na perspectiva de que suscite novos posicionamentos de pesquisadores, profissionais e gestores. Seus comentários ampliam questões centrais do artigo ^1^ e ajudam a deslocar o debate de por que, para como, onde, por quem e para que institucionalizar. Isso é relevante porque evidencia que a consolidação de uma política de avaliação não decorre apenas de sua inscrição nas estruturas do Estado, mas da capacidade de produzir práticas sustentáveis, distribuídas entre os diferentes níveis do sistema e conectadas às necessidades sociais.

A distinção proposta por Matthew Harris ^2^ entre institucionalizar a avaliação da APS e pela APS é instigante. A primeira remete a políticas, estruturas, financiamento, sistemas de informação e avaliações capazes de responder a questões sobre o desempenho e os efeitos da APS. A segunda foca nas equipes, gestores e comunidades, interrogando sua capacidade de compreender as necessidades, analisar criticamente as informações e produzir conhecimento sobre as próprias práticas, incluindo a sua relação com os demais níveis de atenção. Não se trata de escolher entre essas perspectivas. Ao contrário, a institucionalização somente adquire densidade quando ambas se encontram.

Essa distinção explicita um problema recorrente: produzir dados não significa promover avaliação.

A experiência da APS ampliou a disponibilidade de dados, conforme ressalta Marly M. Cruz ^3^, mas a sua transformação em inteligência permanece desigual. Como observa Harris ^2^, uma cultura avaliativa pressupõe curiosidade, capacidade metodológica e disposição para interrogar os resultados e modificar práticas. Portanto, institucionalizar a avaliação exige investir, simultaneamente, em estruturas nacionais e subnacionais e em capacidade avaliativa, incorporando formação em avaliação nos processos de educação permanente, residências, formação dos agentes comunitários e demais profissionais. Além, de uma agenda de pesquisa avaliativa que atenda às lacunas identificadas.

Esse argumento encontra ressonância na contribuição de Ana Luiza d’Ávila Viana ^4^: uma política de avaliação de um sistema multinível precisa ser também multinível. Embora tenhamos destacado a governança tripartite como atributo de uma Política Nacional de Avaliação, Viana ^4^ nos instiga a avançar: é preciso construir uma arquitetura federativa da avaliação, na qual responsabilidades, recursos, capacidades e usos sejam negociados entre níveis de governo e articulados às instâncias de pactuação e controle social.

Isso implica reconhecer que a institucionalização não pode ser concebida como processo linear de difusão de diretrizes do nível federal para os territórios. Os entes federados apresentam capacidades institucionais profundamente desiguais, como regiões, municípios e equipes enfrentam necessidades distintas. Instâncias permanentes de governança, participação das universidades e redes colaborativas, circulação dos resultados e mecanismos de cooperação interfederativa podem reduzir essa assimetria e transformar a avaliação em prática compartilhada de governança.

Aqui torna-se necessário destacar a incongruência existente entre as *Portarias Ministeriais GM/MS nº 3.201* ^5^ e *nº 8.585* ^6^ que tratam, respectivamente, da instituição do Comitê Consultivo de Monitoramento e Avaliação do Sistema Único de Saúde (CCMA-SUS) e da Política de Monitoramento e Avaliação (PMA) no âmbito do Ministério da Saúde. Embora uma delas, mesmo com inconsistência interna, se refira ao SUS, ambas desconsideram o seu caráter interfederativo e tripartite. Se esse pressuposto estiver correto, é preciso corrigir a governança dessas normas que sinalizam uma leitura limitada da Lei Orgânica da Saúde. Nos parece que para o seu cumprimento e, em respeito aos princípios constitucionais de Integralidade, Descentralização e Participação Social, é necessário rever a composição do Comitê, restrito a integrantes do Ministério da Saúde, e o alinhamento de escopo dessas normativas. Por outro lado, as diretrizes constitucionais sinalizam claramente para a necessidade de um olhar atualizado às transformações ocorridas no sistema de saúde e na sociedade quase 40 anos após a sua formulação. Mudanças que demandam uma PMA para o SUS, com pactuação tripartite e submissão ao controle social.

Entretanto, podemos e devemos avançar mais. A contribuição de Luis Augusto Coelho Pisco ^7^ introduz outra fronteira necessária. Se a avaliação integra uma arquitetura democrática de governança, sua institucionalização não pode ignorar a configuração público-privada do sistema de saúde brasileiro. Uma política comprometida com universalidade e equidade deve perguntar não apenas o que e como avaliar, mas também quem é avaliado, por quem e sob quais responsabilidades públicas. A assimetria entre mecanismos de avaliação e responsabilização aplicados ao SUS e aqueles incidentes sobre prestadores e organizações privadas pode produzir uma responsabilização desproporcional do setor público. Ampliar essa discussão significa situar a avaliação no interior da própria regulação do sistema e reafirmar que interesses privados que interferem nas condições de acesso, continuidade e integralidade também precisam integrar o campo de visibilidade da avaliação.

Essa questão remete ao paradoxo que discutimos: a institucionalização fortalece a avaliação, mas pode, simultaneamente, domesticá-la ^1^. Indicadores, metas, certificações e incentivos financeiros podem favorecer transparência e responsabilização, mas também converter a avaliação em dispositivo burocrático de conformidade ou legitimação de decisões previamente estabelecidas. Por isso, institucionalizar implica também criar condições para a independência, a pluralidade e a capacidade crítica da avaliação, garantindo transparência, participação social, diversidade de abordagens, estabilidade de financiamento e espaços públicos de discussão dos resultados para que eles efetivamente influenciem as decisões.

Nessa direção, ganha força a reflexão de Cruz ^3^ sobre a transformação digital e a ampliação da capacidade de produzir informações. Se por um lado, a proposição de Pisco ^7^ pode ser aqui favorecida, por outro, infraestrutura desigual, baixa interoperabilidade e uso burocrático dos sistemas de informação podem reproduzir ou aprofundar as desigualdades. A pergunta central deixa de ser quanto de dados se produz e passa a ser que conhecimento produzimos, para quem e com quais consequências. Novas tecnologias devem fortalecer a aprendizagem, a participação social e as decisões orientadas pela equidade, e não apenas ampliar a capacidade de mensuração e controle.

Nesse ponto também está situada a resiliência, ressaltada por Viana ^4^. Mais do que capacidade de retornar ao estado anterior após uma crise, interessa-nos uma resiliência capaz de produzir aprendizagem e transformação. Mudanças climáticas, emergências sanitárias, violência, desigualdades raciais e sociais, e precarização das condições de vida desafiam uma avaliação centrada exclusivamente no desempenho dos serviços. Avaliar requer compreender como sistemas, instituições e territórios respondem, aprendem e se transformam diante desses processos.

As contribuições dos debatedores permitem delinear mais concretamente a política cuja necessidade defendemos. Precisaria articular governança federativa e capacidade avaliativa; monitoramento e produção de inteligência; *accountability* e aprendizagem; estabilidade institucional e independência crítica; transformação digital e equidade; conhecimento científico e saber social. Talvez, nessa articulação, se encontre o sentido mais profundo da institucionalização. Não pretendemos que a avaliação ocupe simplesmente mais um lugar na burocracia do Estado. Pretendemos que ela se torne uma capacidade permanente do sistema de saúde brasileiro de interrogar a si próprio, dialogar com a sociedade, aprender com os seus territórios e transformar as suas práticas. Institucionalizar, nessa perspectiva, não significa estabilizar a avaliação para evitar mudanças. Significa criar condições para que ela permaneça suficientemente estável para continuar produzindo mudanças.

## Data Availability

Não se aplica.
